# Concurrent chemoradiotherapy versus radiotherapy alone after induction chemoimmunotherapy for stage III NSCLC patients who did not undergo surgery: a single institution retrospective study

**DOI:** 10.1186/s13014-023-02305-5

**Published:** 2023-07-25

**Authors:** Song Guan, Kai Ren, Xuyu Zhang, Meng Yan, Xue Li, Lujun Zhao

**Affiliations:** 1 Department of Radiation Oncology, Tianjin Medical University Cancer Institute & Hospital, National Clinical Research Center for Cancer, Tianjin, China; 2 Tianjin’s Clinical Research Center for Cancer, Tianjin, China; 3 Key Laboratory of Cancer Prevention and Therapy, Tianjin, China

**Keywords:** Non-small cell lung cancer, Concurrent chemoradiotherapy, Immunotherapy

## Abstract

**Background:**

With remarkable success and few side effects, induction chemoimmunotherapy has been used to improve the prognosis of patients with resectable or potentially resectable non-small cell lung cancer (NSCLC), even in stage III disease. However, for patients who are medically inoperable, unresectable or refuse surgery after induction chemoimmunotherapy, it is unclear whether patients should be treated with concurrent chemoradiotherapy (cCRT) or radiotherapy (RT) alone considering patient safety and tolerability. This study aimed to determine whether cCRT is safe and superior to RT alone after chemoimmunotherapy for stage III NSCLC.

**Methods:**

Patients diagnosed with stage III NSCLC who received chemoimmunotherapy followed by cCRT/RT alone without surgery at Tianjin Cancer Hospital between November 2018 to December 2021 were retrospectively collected. Patients were divided into two groups: induction chemoimmunotherapy followed by cCRT (cCRT cohort) or RT alone (RT alone cohort). Kaplan-Meier method was used to estimate survival. Univariate and multivariate Cox regression models were adopted to estimate risk factors for PFS.

**Results:**

Sixty-five patients were included, with 44 (67.7%) received RT alone and 21 (32.3%) received cCRT. Patients in the cCRT group had significantly prolonged PFS (HR = 0.155, p = 0.004), LPFS (HR = 0.225, p = 0.029) and DMFS (HR = 0.028, p = 0.006) than those in the RT alone group. Albeit nonsignificant, a trend toward improved OS (HR = 0.030, p = 0.069) was also observed in the cCRT group. The multivariate analysis further confirmed that cCRT (HR = 0.141, p = 0.008) was the independent factor for promoting a favorable PFS. Treatment-related adverse events were similar between groups (p > 0.05). Patients with consolidation immunotherapy exhibited a trend of improved PFS (HR = 0.398, p = 0.274) and numerically better OS (HR = 0.018, p = 0.209) compared with those without.

**Conclusions:**

For patients with unresectable stage III NSCLC, cCRT following chemoimmunotherapy appears to be safe and may prolong survival compared with radiotherapy alone. Further investigations on the combination of chemoimmunotherapy and CRT are warranted.

**Supplementary Information:**

The online version contains supplementary material available at 10.1186/s13014-023-02305-5.

## Background

Non-small cell lung cancer (NSCLC) constitutes more than 80% of all lung cancers, and approximately one third of which are diagnosed with locally advanced unresectable disease [1, 2]. According to the primary tumor extension and nodal involvement, stage III NSCLC was divided into two subgroups: potentially resectable and unresectable disease [3]. For patients with unresectable stage III, the standard of care is concurrent chemoradiotherapy (cCRT) followed by consolidation immunotherapy with PD-L1 inhibitor durvalumab based on the practicing-changing results of the PACIFIC trial [4]. For patients with resectable stage III NSCLC, both adjuvant and neoadjuvant chemotherapy had a limited survival benefit [5, 6]. Until recent years, robust evidence has shown that combining immunotherapy and surgery could provide tremendous potential benefits [7–10]. With the remarkable success and few side effects, the use of immunotherapy is likely to be extended to earlier stages of the disease, including potentially resectable stage III [8–10].

Currently, neoadjuvant chemoimmunotherapy accounts for the majority of neoadjuvant immunotherapy clinical trials and generally reports an improved pathological response compared with neoadjuvant immunotherapy alone, thereby improving prognosis [8–14]. The CheckMate-816 trial, which enrolled patients with stage IB to IIIA resectable NSCLC, was the first phase 3 clinical trial to demonstrate a survival benefit of induction chemoimmunotherapy [8]. While for patients who are medically inoperable, unresectable or refuse surgery after induction chemoimmunotherapy, chemoradiotherapy (CRT) is the backbone of radical treatment and appears to be the first choice. However, there is still lacking evidence to guide whether patients should receive cCRT or radiotherapy (RT) alone, taking into account patient safety and tolerability. Previous studies have shown that the combination of radiotherapy (RT) with immunotherapy increases the incidence of pneumonitis, as well as radiation recall pneumonitis [15–17]. Additionally, radiotherapy concurrent with chemotherapy significantly increased the incidence of acute toxicity than radiotherapy (RT) alone [18–20]. However, the safety of cCRT following induction chemoimmunotherapy is still unclear. Herein, to determine whether cCRT is safe and superior to RT alone after induction chemoimmunotherapy in stage III NSCLC patients who did not undergo surgery, we performed this retrospective study.

## Methods

### Population

Patients with unresectable or potentially resectable stage III NSCLC who received chemoimmunotherapy followed by cCRT/RT alone without surgery at Tianjin Cancer Hospital from November 2018 to December 2021 were retrospectively collected. Patients with history of any cancer-specific treatment, received concurrent immunotherapy, induction immunotherapy alone or received immunotherapy as part of a clinical trial were excluded. Patients were divided into 2 groups according to the CRT modality they received: (1) cCRT group: induction chemoimmunotherapy followed by cCRT; (2) RT alone group: induction chemoimmunotherapy followed by RT alone. The CRT modality for patients was determined according to local practice based on performance status, patient tolerance, cycles of induction chemoimmunotherapy, etc.

Baseline demographic, clinical, and pathologic data were abstracted from patients’ electronic health records. NSCLC histology was classified according to WHO criteria [21]. Disease staging was based on the eighth edition of the American Joint Committee on Cancer and International Union Against Cancer TNM stage classification for lung cancer [22]. The age-adjusted Charlson comorbidity index (ACCI) was used to assess the comorbidities between groups [23]. The cut-off point was determined by the median score.

This study conformed to the provisions of the Declaration of Helsinki (as revised in 2013). The institutional review board of Tianjin Medical University Cancer Institute and Hospital gave ethical approval for this study.

### Drug treatment

All patients were treated with immunotherapy using ICIs, such as sintilimab, pembrolizumab, durvalumab, camrelizumab, tislelizumab or nivolumab. These six kinds of PD-1/PD-L1 inhibitors have been approved for the treatment of NSCLC, and have shown encouraging efficacy in NSCLC patients [24]. The chemotherapy regimen for patients was determined according to local practice based on the performance status, corresponding pathological type of the tumor, etc., including platinum-based doublet chemotherapy, mono-chemotherapy.

### Study outcomes

The clinical outcomes assessed in this study were PFS, LPFS, DMFS and OS. Progression-free survival (PFS) was calculated from the date of beginning induction treatment to the date of progression, death from any cause, or last follow-up, whichever came first. Local progression-free survival (LPFS) was calculated from the date of beginning induction treatment to the date of local progression or death by any cause. Distant metastasis-free survival (DMFS) was defined as the time from induction treatment to distant metastasis or death by any cause. Overall survival (OS) was defined as the time from the date of beginning induction treatment to the date of death from any cause, or last follow-up. The response after induction treatment including complete response (CR), partial response (PR), stable disease (SD) and progressive disease (PD) were assessed. Treatment-related adverse events (TRAEs) owing to treatment were classified according to the Common Terminology Criteria for Adverse Events version 5.0 (CTCAE 5.0).

### Statistical analysis

Patient characteristics were evaluated between treatment groups using Fisher’s exact test for categorical variables. Kaplan-Meier survival curves were generated to estimate survival across treatment groups, and the log-rank test was used for subgroup comparisons. When a p-value of ≤ 0.15 was obtained from the univariate analysis, the factor was selected for multivariate Cox regression analyses. In the subgroup analysis, propensity-matched (PSM) analysis was performed. Statistical significance was declared when a p-value of < 0.05. All statistical analyses were performed using SPSS, version 25.0 (SPSS, Chicago, USA).

## Results

### Baseline characteristics

A total of 65 consecutive patients were included, with 44 (67.7%) in the RT alone group and 21 (32.3%) in the cCRT group, respectively. Baseline characteristics are presented in Table 1. The median age was 62 years (range 27–77), and most patients were male, never smoked and Eastern Cooperative Oncology Group (ECOG) performance status 1. The WHO histology was predominantly squamous carcinoma, and most patients had stage IIIA or IIIB. Five patients with lung adenocarcinoma had unknown driver gene status, the rest were all driver gene wild-type. Adverse events of any grade (34.1% in the RT alone group vs. 28.6% in the cCRT group, p = 0.656) and grade 3/4 (9.1% in the RT alone group vs. 4.8% in the cCRT group, p = 0.909) after induction chemoimmunotherapy were similar between groups. The baseline characteristics of patients in the two treatment groups were well balanced.

**Table 1:** The baseline demographic and clinical characteristics of patients

Characteristic	RT alone (n = 44)		cCRT (n = 21)		
	n	%	n	%	p
Age					
< 65	26	59.1	16	76.2	0.178
≥ 65	18	40.9	5	23.8	
Sex					
Male	39	88.6	18	85.7	1.000
Female	5	11.4	3	14.3	
WHO histology					
Squamous	30	68.2	14	66.7	0.364
Non-squamous	12	27.3	4	19.0	
NOS	2	4.5	3	14.3	
Stage					
IIIA	16	36.4	10	47.6	0.623
IIIB	24	54.5	10	47.6	
IIIC	4	9.1	1	4.8	
Radiation dose					
<54 Gy	3	6.8	1	4.8	1.000
≥54 Gy	41	93.2	20	95.2	
Smoking history					
Never smoked	7	15.9	4	19.0	1.000
Former or current	37	84.1	17	81.0	
ECOG					
0	3	6.8	1	4.8	0.891
1	38	86.4	19	90.5	
2	3	6.8	1	4.8	
Consolidation ICI					
No	34	77.3	16	76.2	1.000
Yes	10	22.7	5	23.8	
ACCI					
≤ 2	23	52.3	11	52.4	0.993
> 2	21	47.7	10	47.6	
Response					
PR + CR	29	65.9	11	52.4	0.294
SD + PD	15	34.1	10	47.6	

### Treatment

All patients received induction chemoimmunotherapy, with a median of 4 cycles of immunotherapy (rang 1–9) and chemotherapy (rang 2–8), respectively. 31 (47.7%) patients were treated with sintilimab, 15 (23.1%) with pembrolizumab, 1 (1.5%) with durvalumab, 9 (13.8%) with camrelizumab, 6 (9.2%) with tislelizumab, 3 (4.6%) with nivolumab. Sixty patients remained unresectable and 5 patients refused surgery after induction chemoimmunotherapy, including 3 in the cCRT group and 2 in the RT alone group. A total of 44 patients in the RT alone cohort received a median of 4 cycles of induction immunotherapy (range 1–9) and induction chemotherapy (range 2–8). While in the cCRT cohort, 21 patients received a median of 3 cycles of induction immunotherapy (range 1–4) and induction chemotherapy (range 2–7). In addition, 15 patients further received consolidation immunotherapy, including 10 patients (22.7%) in the RT alone group and 5 patients (23.8%) in the cCRT group.

Among the 21 patients who received cCRT, most patients (16 of 21, 76.2%) received platinum-based doublet chemotherapy, while the rest were all received monochemotherapy, including 3 (14.3%) patients with weekly albumin-bound paclitaxel, and one each with weekly nedaplatin and pemetrexed alone.

### Survival analysis

In the entire cohort, the median follow-up time was 14.8 months (range 5.5–41.8 months) after treatment initiation. Median PFS was 23.9 months, and OS data were immature at date cutoff. A total of 26 patients (40.0%) experienced progression, including 24 (54.5%) patients in the RT alone group and 2 (9.5%) patients in the cCRT group. Of these patients, 10 patients had local relapse only, including 2 in the cCRT group and 8 in the RT alone group, 7 had distant metastasis, and 9 had both and distant relapse. While 9 patients had died at the time of analysis, all of them were in the RT alone group.

**Fig. 1:**
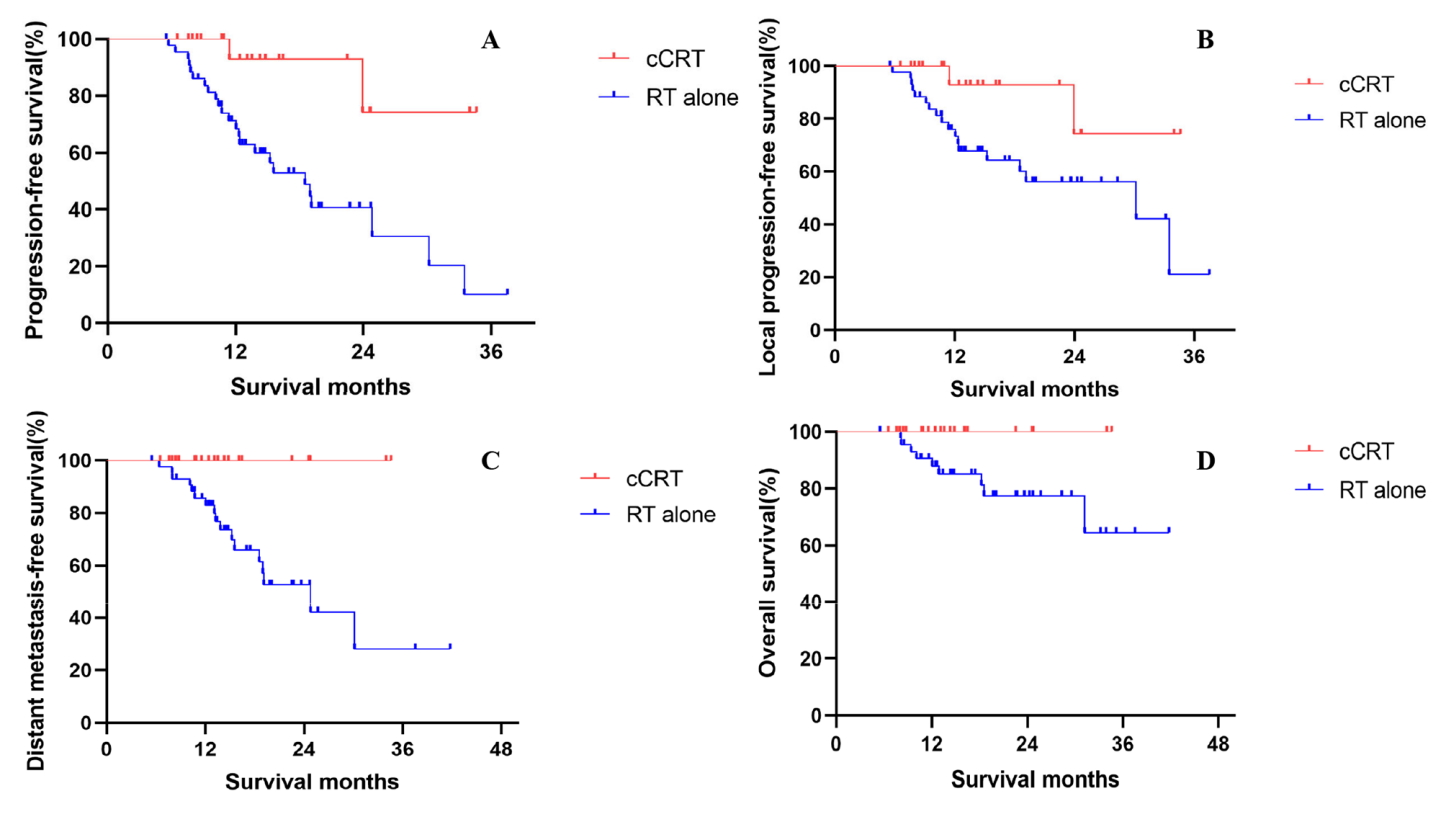
PFS (**A**), LPFS (**B**), DMFS (**C**) and OS (**D**) between the two treatment groups

Median PFS was 18.5 months in the RT alone group vs. not reached (NR) in the cCRT group, with a 1-year PFS rate of 71.2% vs. 92.9% and a 2-year PFS rate of 40.7% vs. 74.3% (HR = 0.155, p = 0.004, Fig. 1A). Median LPFS was 30.2 months in the RT alone group vs. NR in the cCRT group, with a 1-year LPFS rate of 76.0% vs. 92.9% and a 2-year LPFS rate of 56.2% vs. 74.3% (HR = 0.225, p = 0.029, Fig. 1B). Median DMFS and OS were immature in both treatment groups, with a 2-year DMFS rate of 52.7% for the RT alone group compared with 100.0% for the cCRT group (HR = 0.028, p = 0.006, Fig. 1C), and a 2-year OS rate of 77.3% for the RT alone group compared with 100.0% for the cCRT group (HR = 0.030, p = 0.069, Fig. 1D).

### Multivariate analysis for PFS

For the whole population, we further explored the risk factors affecting PFS. The multivariate Cox regression analysis revealed that cCRT (HR = 0.141, p = 0.008) and elder (HR = 0.357, p = 0.039) were the independent factors for promoting a favorable PFS (Table 2).

**Table 2:** Univariate and multivariate Cox regression analyses for PFS

Factor	Univariate			Multivariate		
	HR	95% CI	p	HR	95% CI	p
Age						
< 65	1.000 (reference)			1.000 (reference)		
≥ 65	0.409	0.154,1.089	0.073	0.357	0.134,0.951	**0.039**
Sex						
Male	1.000 (reference)					
Female	0.508	0.119,2.159	0.359			
WHO histology						
Squamous	1.000 (reference)					
Non-squamous	0.726	0.303,1.739	0.472			
NOS	0.000	0.000	0.979			
Stage						
IIIA	1.000 (reference)					
IIIB	0.964	0.416,2.233	0.932			
IIIC	2.341	0.628,8.732	0.205			
Radiation dose						
<54 Gy	1.000 (reference)					
≥54 Gy	0.591	0.172,2.031	0.403			
Smoking history						
Never smoked	1.000 (reference)					
Former or current	1.372	0.462,4.078	0.569			
ECOG						
0	1.000 (reference)					
1	2.155	0.286,16.260	0.457			
2	0.000	0.000	0.980			
Consolidation ICI						
No	1.000 (reference)					
Yes	0.888	0.355,2.219	0.799			
ACCI						
≤ 2	1.000 (reference)					
> 2	0.710	0.325,1.553	0.392			
Response						
PR + CR	1.000 (reference)					
SD + PD	1.176	0.533,2.598	0.688			
Treatment						
RT alone	1.000 (reference)			1.000 (reference)		
cCRT	0.155	0.037,0.659	**0.012**	0.141	0.033,0.600	**0.008**

### Treatment-related adverse events

Twenty patients (69.0%) in the RT alone group and 8 patients (53.3%) in the cCRT group developed any grade of pneumonitis, respectively. Grade 3 or higher pneumonitis occurred in 2 (6.9%) and 0 (0.0%) patients in the RT alone and cCRT groups, respectively. In addition, 1 patient of each had concurrent fungal pneumonia and grade 2 dermatitis in the RT alone group, and 2 patients developed peripheral neurotoxicity in the cCRT group. The remaining documented TRAEs are shown in Table 3.

**Table 3:** TRAEs between the two treatment groups

TRAE	RT alone		cCRT		
	n	%	n	%	p
Pneumonitis	31	72.1	14	63.6	0.485
G3/4 pneumonitis	5	11.6	0	0.0	0.241
Esophagitis	7	16.3	2	9.1	0.679
G3/4 esophagitis	0	0.0	0	0.0	NA
Hematologic toxicity	28	65.1	17	77.3	0.315
G3/4 hematologic toxicity	6	14.0	8	36.4	0.078

### Subgroup analysis

**Fig. 2:**
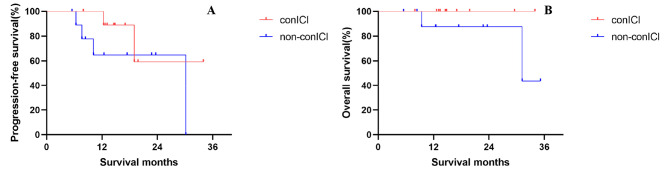
PFS (**A**) and OS (**B**) between patients with and without consolidation immunotherapy after PSM

A total of 15 patients further received consolidation immunotherapy. Median PFS was 19.0 months for patients with consolidation immunotherapy compared with 24.8 months for those without, with a 2-year PFS rate of 43.9% vs. 50.1% (HR = 0.888, p = 0.799, Fig. S1A in the supplementary material). Median OS was NR in either group, the 2-year OS rate was 85.7% for patients with consolidation immunotherapy compared with 82.2% for those without (HR = 0.994, p = 0.994, Fig. S1B). After PSM (Table S1 in the Supplementary material), median PFS was NR for patients with consolidation immunotherapy compared with 30.2 months for patients without, with a 2-year PFS rate of 59.3% vs. 64.8% (HR = 0.398, p = 0.274, Fig. 2A). Median OS was still NR in either group, the 2-year OS rate was 100.0% for patients with consolidation immunotherapy compared with 87.5% for those without (HR = 0.018, p = 0.209, Fig. 2B).

## Discussion

Although cCRT followed by consolidation immunotherapy is the current standard of care for patients with unresectable stage III NSCLC, with the gradual application of immunotherapy in the neoadjuvant setting, an increasing number of patients who are medically inoperable, unresectable or refuse surgery after receiving induction chemoimmunotherapy are switching to CRT. However, the choice of cCRT or RT alone remains unclear. In this study, we confirmed the safety and efficacy of cCRT after chemoimmunotherapy for patients with stage III NSCLC. To our knowledge, this is the first study on the choice of cCRT vs. RT alone after induction chemoimmunotherapy for patients with stage III NSCLC.

More recently, due to the higher rate of pathological response and even the ability to improve prognosis, induction chemoimmunotherapy has become a hot topic in clinical research. However, for patients who are medically inoperable, unresectable or refuse surgery after receiving chemoimmunotherapy, CRT appears to be the first choice. Nevertheless, there is still no relevant study on which modality of CRT to choose. Before the era of immunotherapy, cCRT demonstrated superiority to sequential treatment in patients with stage III disease in terms of OS and reduced risk of locoregional progression, but at the cost of increased acute toxicity [18–20]. While in the immunotherapy era, given that patients have already received several cycles of chemoimmunotherapy, data is still lacking to support whether subsequent cCRT is safe, tolerable and still provides survival benefits. Our result showed that patients who received cCRT after induction chemoimmunotherapy had prolonged survival without significantly increasing the incidence of TRAEs, except that hematological toxicity seemed more common. Besides, the multivariate analysis further confirmed that cCRT was the independent factor for a prolonged PFS, suggesting the importance of cCRT even in the immunotherapy era. Notably, our study showed that elder was an independent factor favoring PFS, which is inconsistent with previous studies [25, 26]. This may be related to the fact that most of elderly patients in this study had earlier TNM staging (IIIA: 52.2% of ≥ 65 years vs. 33.3% of < 65 years).

Currently, whether consolidation immunotherapy combined with induction immunotherapy could provide further survival benefits is still unclear. Although the AFT-16 trial, which administered single-agent immunotherapy before and after CRT, and the KEYNOTE-799 trial, which used chemoimmunotherapy before CRT followed by ICI during and after CRT, showed high ORRs and prolonged survival, both were single-arm studies [27–29]. Our study demonstrated that patients with induction and consolidation immunotherapy exhibited a trend of improved PFS and numerically better OS compared with induction immunotherapy alone, which is similar to the results of Wang et al. (median PFS 23.8 months vs. 21.9 months, 2-year OS 85.8% vs. 64.2%) [30]. Taken together, these findings indicate that the combination of induction and consolidation immunotherapy appears to be more beneficial to patients, which requires prospective clinical trials to further confirm.

There are some limitations in our study. First, this is a retrospective study conducted at a single institution, which may limit the generalizability of these results. In addition, due to the relatively shorter follow-up time and relatively small sample size, the OS outcome was immature which limit we further exploring the exact role of different CRT modality on prognosis. Moreover, PD-L1 expression and driver-gene mutation status were detected only in part of patients, which may lead to analysis bias. Meanwhile, although previous study [31] showed that there is no significant difference in the efficacy of different ICIs, the identical ICI agent should be used to analyze its exact impact on prognosis to avoid biased results as much as possible in future studies. Certainly, prospective and longer follow-up trials are needed to draw definitive conclusions. Despite these limitations, this is the first study to compare the efficacy and safety of cCRT and radiotherapy alone for patients with stage III NSCLC after receiving induction chemoimmunotherapy to our knowledge, and we believe that this study could suggest a new direction for research or provide some clinical references.

## Conclusions

In conclusion, for patients with unresectable stage III NSCLC, cCRT following chemoimmunotherapy appears to be safe and may prolong survival compared with radiotherapy alone. Further investigations on the combination of chemoimmunotherapy and CRT are warranted.

## Electronic supplementary material

Below is the link to the electronic supplementary material.

Supplementary Material 1

## Data Availability

The datasets used and analyzed during the current study are available from the corresponding author on reasonable request.

## Abbreviations

NSCLC: Non-small cell lung cancer
cCRT: Concurrent chemoradiotherapy
RT: Radiotherapy
ACCI: Age-adjusted Charlson comorbidity index
PFS: Progression-free survival
LPFS: Local progression-free survival
DMFS: Distant metastasis-free survival
OS: Overall survival
CR: Complete response
PR: Partial response
SD: Stable disease
PD: Progressive disease
NR: Not reached
ICIs: Immune checkpoint inhibitors
TRAEs: Treatment-related adverse events
PSM: Propensity-matched analysis
HRs: Hazard ratios

## References

[1] Crino L, Weder W, van Meerbeeck J (2010). Early stage and locally advanced (non-metastatic) non-small-cell lung cancer: esmo clinical practice guidelines for diagnosis, treatment and follow-up. Ann Oncol.

[2] Govindan R, Bogart J, Vokes EE (2008). Locally advanced non-small cell lung cancer: the past, present, and future. J Thorac Oncol.

[3] Goldstraw P, Chansky K, Crowley J (2016). The IASLC Lung Cancer Staging Project: proposals for revision of the TNM Stage Groupings in the Forthcoming (Eighth) Edition of the TNM classification for Lung Cancer. J Thorac Oncol.

[4] Antonia SJ, Villegas A, Daniel D (2017). Durvalumab after chemoradiotherapy in stage III non-small-cell lung cancer. N Engl J Med.

[5] Watanabe SI, Nakagawa K, Suzuki K (2017). Neoadjuvant and adjuvant therapy for stage III non-small cell lung cancer. Jpn J Clin Oncol.

[6] Burdett S, Stewart LA, Rydzewska L (2006). A systematic review and meta-analysis of the literature: chemotherapy and surgery versus surgery alone in non-small cell lung cancer. J Thorac Oncol.

[7] Felip E, Altorki N, Zhou C (2021). Adjuvant atezolizumab after adjuvant chemotherapy in resected stage IB-IIIA non-smallcell lung cancer (IMpower010): a randomised, multicentre, open-label, phase 3 trial. Lancet.

[8] Forde PM, Spicer J, Lu S (2022). Neoadjuvant Nivolumab plus Chemotherapy in Resectable Lung Cancer. New Engl J Med.

[9] Provencio M, Nadal E, Insa A (2020). Neoadjuvant chemotherapy and nivolumab in resectable non-small-cell lung cancer (NADIM): an open-label, multicentre, single-arm, phase 2 trial. Lancet Oncol.

[10] Rothschild SI, Zippelius A, Eboulet EI, Swiss Group for Clinical Cancer Research (SAKK) (2021). SAKK 16/14: durvalumab in addition to neoadjuvant chemotherapy in patients with stage IIIA(N2) non-small-cell lung cancer-a multicenter single-arm phase II trial. J Clin Oncol.

[11] Jiang J, Wang Y, Gao Y (2022). Neoadjuvant immunotherapy or chemoimmunotherapy in non-small cell lung cancer: a systematic review and meta-analysis. Transl Lung Cancer R.

[12] Cascone T, William WNJr., Weissferdt A (2021). Neoadjuvant nivolumab or nivolumab plus ipilimumab in operable non-small cell lung cancer: the phase 2 randomized NEOSTAR trial. Nat Med.

[13] Tong BC, Gu L, Wang X (2022). Perioperative outcomes of pulmonary resection after neoadjuvant pembrolizumab in patients with nonsmall cell lung cancer. J Thorac Cardiovasc Surg.

[14] Zhao ZR, Yang CP, Chen S (2021). Phase 2 trial of neoadjuvant toripalimab with chemotherapy for resectable stage III non-small-cell lung cancer. Oncoimmunology.

[15] Shaverdian N, Beattie J, Thor M (2020). Safety of thoracic radiotherapy in patients with prior immune-related adverse events from immune checkpoint inhibitors. Ann Oncol.

[16] Aiad M, Fresco K, Prenatt Z (2022). Comparison of Pneumonitis Rates and Severity in patients with Lung Cancer treated by Immunotherapy, Radiotherapy, and immunoradiotherapy. Cureus.

[17] Cousin F, Desir C, Ben Mustapha S (2021). Incidence, risk factors, and CT characteristics of radiation recall pneumonitis induced by immune checkpoint inhibitor in lung cancer. Radiother Oncol.

[18] Aupérin A, Le Péchoux C, Rolland E (2010). Meta-analysis of concomitant versus sequential radiochemotherapy in locally advanced non-small-cell lung cancer. J Clin Oncol.

[19] Curran WJ, Paulus R, Langer CJ (2011). Sequential vs. concurrent chemoradiation for stage III non-small cell lung cancer: randomized phase III trial RTOG 9410. JNCI-Jnatl Cancer I.

[20] O’Rourke N, Roqué I, Figuls M, Farré Bernadó N et al. Concurrent chemoradiotherapy in non-small cell lung cancer. Cochrane Database Syst Rev. 2010: CD002140. 10.1002/14651858.CD002140.pub3 PMC12584098 20556756

[21] Travis WD, Brambilla E, Nicholson AG (2015). The 2015 World Health Organization classification of lung tumors: impact of genetic, clinical and radiologic advances since the 2004 classification. J Thorac Oncol.

[22] Detterbeck FC, Boffa DJ, Kim AW (2017). The eighth edition lung cancer stage classification. Chest.

[23] Yang CC, Fong Y, Lin LC (2018). The age-adjusted Charlson comorbidity index is a better predictor of survival in operated lung cancer patients than the Charlson and Elixhauser comorbidity indices. Eur J Cardio-thorac.

[24] Gan J, Huang Y, Fang W, Zhang L (2021). Research progress in immune checkpoint inhibitors for lung cancer in China. Ther Adv Med Oncol.

[25] Grossi F, CrinòL, Logroscino A (2018). Use of nivolumab in elderly patients with advanced squamous non-small-cell lung cancer: results from the italian cohort of an expanded access programme. Eur J Cancer.

[26] Galli G, De Toma A, Pagani F (2019). Efficacy and safety of immunotherapy in elderly patients with non-small cell lung cancer. Lung Cancer.

[27] Ross HJ, Kozono DE, Urbanic JJ (2021). AFT-16: phase II trial of neoadjuvant and adjuvant atezolizumab and chemoradiation (CRT) for stage III non-small cell lung cancer (NSCLC). J Clin Oncol.

[28] Jabbour SK, Lee KH, Frost N (2021). Pembrolizumab Plus Concurrent Chemoradiation Therapy in patients with Unresectable, locally Advanced, Stage III Non-Small Cell Lung Cancer: the phase 2 KEYNOTE-799 nonrandomized trial. Jama Oncol.

[29] Reck M, Lee KH, Frost N (2022). Two-year update from KEYNOTE-799: Pembrolizumab plus concurrent chemoradiation therapy (cCRT) for unresectable, locally advanced. stage III NSCLC.

[30] Wang Y, Zhang T, Wang J et al. Induction Immune Checkpoint inhibitors and chemotherapy before definitive chemoradiation therapy for patients with Bulky Unresectable Stage III non-small-cell Lung Cancer. Int J Radiat Oncol Biol Phys. 2023-01–6. 10.1016/j.ijrobp.2022.12.042 36623605

[31] Miao K, Zhang X, Wang H (2022). Real-World Data of different Immune checkpoint inhibitors for Non-Small Cell Lung Cancer in China. Front Oncol.

