# Supplementary material for: Concurrent chemoradiotherapy versus radiotherapy alone after induction chemoimmunotherapy for stage III NSCLC patients who did not undergo surgery: a single institution retrospective study
Source: Radiat Oncol. 2023 Jul 25;18:122. doi: 10.1186/s13014-023-02305-5 (PMC10367242; doi:10.1186/s13014-023-02305-5)
Supplement: Supplementary file 1 — Supplementary Material 1 [file 13014_2023_2305_MOESM1_ESM.docx]

**Table S1** The baseline demographic and clinical characteristics of patients after PSM

| Characteristic | non-conICI (n=10) | | conICI (n=10) | |  |
| --- | --- | --- | --- | --- | --- |
|  | n | % | n | % | p |
| Age |  |  |  |  |  |
| <65 | 6 | 60.0 | 6 | 60.0 | 1.000 |
| ≥65 | 4 | 40.0 | 4 | 40.0 |  |
| Sex |  |  |  |  |  |
| Male | 9 | 90.0 | 9 | 90.0 | 1.000 |
| Female | 1 | 10.0 | 1 | 10.0 |  |
| WHO histology |  |  |  |  |  |
| Squamous | 7 | 70.0 | 7 | 70.0 | 1.000 |
| Non-squamous | 2 | 20.0 | 2 | 20.0 |  |
| NOS | 1 | 10.0 | 1 | 10.0 |  |
| Stage |  |  |  |  |  |
| IIIA | 3 | 30.0 | 3 | 30.0 | 1.000 |
| IIIB | 6 | 60.0 | 6 | 60.0 |  |
| IIIC | 1 | 10.0 | 1 | 10.0 |  |
| Radiation dose |  |  |  |  |  |
| <54Gy | 2 | 20.0 | 0 | 0.0 | 0.456 |
| ≥54Gy | 8 | 80.0 | 10 | 100.0 |  |
| Smoking history |  |  |  |  |  |
| Never smoked | 2 | 20.0 | 1 | 10.0 | 1.000 |
| Former or current | 8 | 80.0 | 9 | 90.0 |  |
| ECOG |  |  |  |  |  |
| 0 | 1 | 10.0 | 0 | 0.0 | 1.000 |
| 1 | 9 | 90.0 | 10 | 100.0 |  |
| CRT modality |  |  |  |  |  |
| RT alone | 8 | 80.0 | 6 | 60.0 | 0.626 |
| cCRT | 2 | 20.0 | 4 | 40.0 |  |


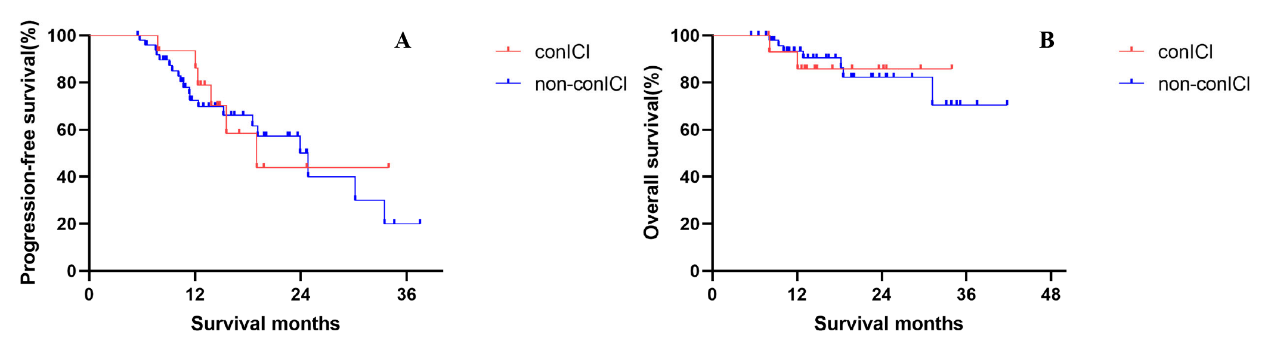


**Fig. S1** PFS (A) and OS (B) between patients with and without consolidation immunotherapy before PSM
